# Color priming in pop-out search depends on the relative color of the target

**DOI:** 10.3389/fpsyg.2014.00289

**Published:** 2014-04-08

**Authors:** Stefanie I. Becker, Christian Valuch, Ulrich Ansorge

**Affiliations:** ^1^School of Psychology, The University of QueenslandBrisbane, QLD, Australia; ^2^Center for Interdisciplinary Research, Bielefeld UniversityBielefeld, Germany; ^3^Cognitive Research Platform, University of ViennaVienna, Austria; ^4^Faculty of Psychology, University of ViennaVienna, Austria

**Keywords:** attention, color, priming, eye movement, pop-out, visual search, priming of pop-out, relational

## Abstract

In visual search for pop-out targets, search times are shorter when the target and non-target colors from the previous trial are repeated than when they change. This *priming effect* was originally attributed to a feature weighting mechanism that biases attention toward the target features, and away from the non-target features. However, more recent studies have shown that visual selection is strongly context-dependent: according to a *relational account* of feature priming, the target color is always encoded relative to the non-target color (e.g., as redder or greener). The present study provides a critical test of this hypothesis, by varying the colors of the search items such that either the relative color or the absolute color of the target always remained constant (or both). The results clearly show that color priming depends on the relative color of a target with respect to the non-targets but not on its absolute color value. Moreover, the observed priming effects did not change over the course of the experiment, suggesting that the visual system encodes colors in a relative manner from the start of the experiment. Taken together, these results strongly support a relational account of feature priming in visual search, and are inconsistent with the dominant feature-based views.

## INTRODUCTION

Visual search is one of the most frequent activities in everyday life, and has been frequently used in research to examine how we allocate attention. One of the first insights of studies using the visual search paradigm was that some searches are slower and effortful, whereas others are quick and efficient, allowing immediate selection of the sought-after target (e.g., Treisman and Gelade, 1980). Among the most efficient searches are so-called pop-out searches, in which the target differs in a single unique feature from the irrelevant non-targets (e.g., a red target among green non-target items). Initially, it was thought that pop-out targets could be found immediately, and with the first glance due to their bottom-up feature contrast alone – without any other factors modulating search performance (e.g., Treisman and Sato, 1990; Wolfe, 1994; Yantis, 2000). However, Maljkovic and Nakayama (1994) later showed that search times in pop-out search are faster when the target and non-target have the same colors as on the previous trial.

In the study of Maljkovic and Nakayama (1994), observers had to search for a pop-out target that was randomly either red and presented among green non-targets, or green among red non-targets. Note that the color contrast of the target was always the same across all trials (red-green), and that the target was the only item in the display with a feature contrast. Yet, repeating the target and non-target colors across trials speeded search, compared to when the target and non-target colors had switched (i.e., from a red to a green target or *vice versa*). These results have been replicated numerous times (e.g., Tanaka and Shimojo, 1996; McPeek et al., 1999; Hillstrom, 2000; Goolsby and Suzuki, 2001; Wolfe et al., 2003; Kristjansson et al., 2007; Becker, 2008a,b,c; Folk and Remington, 2008; Lamy et al., 2008; Leonard and Egeth, 2008). Collectively, these studies show that pop-out search is not completely determined by the color contrast of the actual target, but is modulated by the trial history – with facilitated search when the target color is repeated.

To explain this effect of the previous trial on search performance, Maljkovic and Nakayama (1994) proposed that visual selection of the target on a given trial *primes* or *biases* attention toward similar features, which facilitates selection of the target if the target-defining feature from the previous trial is repeated. In line with current theories of attention (e.g., Treisman, 1988; Wolfe, 1994; Lee et al., 1999; Navalpakkam and Itti, 2006; Nakayama and Martini, 2011), it was moreover proposed that the target and non-target colors are encoded separately and independently of each other, on separate “feature maps.” In these models, different features such as colors are encoded by separate populations of sensory neurons. When a target with a particular feature (e.g., red) is selected, the attentional gain for the target color is enhanced, and the attentional gain for the non-target color can additionally be reduced. These gains would then automatically transfer to the subsequent trial, and bias attention independently toward the previous target feature, and/or away from the previous non-target feature (e.g., Maljkovic and Nakayama, 1994; Kristjansson and Driver, 2008; Lamy et al., 2008).

Recent studies, however, cast doubt on the assumption that feature gain operates independently on the color of the target and the color of the non-targets. Specifically, it has been shown that the target color is not processed independently of the non-target color. Instead, the target color is encoded *relative* to the non-target colors (e.g., as *redder* or *greener*). Plus, it is the *relative color* information that carries over to the next trial and primes selection of items with the same relative color, whereas the actual color values of the target or the distractors do not play such an important role for priming or selection (e.g., Becker, 2010a). This *relational account* of feature priming has so far been established for priming effects in search for color, luminance, size, and shape (Becker, 2010a, 2013).

In the corresponding experiments, observers were typically asked to search for a pop-out target whose features varied differently in two blocked conditions. In one condition, the target or non-target features varied such that their relative features reversed on switch trials (e.g., from smaller target to larger target, or vice versa), but either the target’s physical feature or the non-targets’ physical feature always remained constant (e.g., either target always medium sized, or non-targets always medium sized). In a second condition, both the target’s and non-targets’ physical features could vary, but their relative features remained constant across all trials (e.g., target always smaller; Becker, 2010a, 2013).

According to feature-priming accounts, changing the physical attributes of the target (and maybe of the non-targets) across trials should delay attention shifts to the target and incur costs (compared to repeat trials; henceforth: “switch costs”). By contrast, according to the relational account, changing the physical feature of the target (or maybe also the non-targets) is *not* sufficient to incur costs. Because priming and selection both operate on the relative feature of the target, switch costs should occur only when the relative feature of the target changes (e.g., from larger to smaller, or from redder to non-redder).

Studies that critically tested the predictions of the relational account against the feature-based view have so far supported the relational account: in search for a color, luminance, shape or size target, switch costs were completely independent of whether the physical feature of the target changed or not, and occurred only when the relative feature of the target changed (e.g., Becker, 2010a, 2013). These results suggest that the target feature is always encoded relative to the features of non-targets; contrary to the view that the target and non-target features are processed separately and independently of each other.

However, the evidence for the relational account is still incomplete, especially with regard to color search. This holds because previous studies investigating color priming have only contrasted conditions that always involved a change in the target feature (on switch trials). For example, one study showed that presenting a yellow or orange target among consistently red non-targets in a *steady relation condition* (target always yellower) did not incur switch costs, whereas presenting a yellow or red target among consistently orange non-targets in a *relation reversal condition* (target redder or yellower) produced switch costs (see Becker, 2010a). The results supported the relational account, because switch costs occurred only when the relative color of the target changed, not when its physical color changed. However, the results may still be consistent with a feature-based account. Of note, the two different target colors were more similar to each other (yellow, orange) in the condition in which the relative target color remained the same, compared to the relation reversal condition (target yellow, red). Moreover, the colors were all quite similar to each other, as they did not vary from full red to full yellow, but only from red-orange to yellow–orange (Becker, 2010a). Hence, it seems possible that the two similar target colors in the steady relation condition could be processed via the same color channel or feature map, because they elicited the same responses in one channel, whereas the more dissimilar target colors in the relation reversal condition (red, yellow) could not be processed by the same channel. Hence, the relation reversal condition may have produced switch costs because encoding of the target necessitated a switch between the red and yellow channel whereas the targets in the steady relation condition failed to show switch costs because they were encoded by a single channel.

The aim of the present study to provide a more decisive test of the relational account versus the dominant feature-based accounts of priming. Specifically, we examined whether processing of the target via a single channel versus the necessity to switch between different channels would indeed determine switch costs. To that purpose, priming effects were tested in different conditions of a color search task. In one condition, the relative target color varied while the target’s color value always remained the same, encouraging single channel processing. In another condition, the relative target color was always constant while the target’s and non-targets’ color values varied, such that the non-targets inherited the former target color or vice versa (half-switch; enforcing multiple-channel processing). These conditions are diametrically opposite to the previous studies on color priming, and can thus clarify whether switch costs depend on a change in the target’s relative color, or the need to switch to a different color-processing channel. Moreover, to test whether the results would generalize to different colors, we utilized two different sets of colors – a similar and a dissimilar color set.

Search performance was assessed by monitoring the observer’s eye movements during visual search. In particular, we measured the *search times* as the time from the onset of the search display until the first fixation on the target, and in addition, report priming effects on the first eye movement in a trial. Eye movements are usually preceded by a covert attention shift to the saccade target location, so that the first eye movement on a trial can provide a reliable measure for covert attention shifts (e.g., Hoffman and Subramaniam, 1995; Kowler et al., 1995; Deubel and Schneider, 1996). The first eye movement also occurs about 500 ms prior to a button-press response, and is therefore less affected by processes that are unrelated to visual search proper, such as the perceptual identification of the stimulus and response selection (e.g., Töllner et al., 2012). This means that eye movements can provide a more accurate estimate of early attentional processes than later measures such as the mean response times (RTs), which can be influenced by later response selection processes. Of note, repetitions or changes of the target can also affect later, response selection processes (Huang and Pashler, 2005; Mortier et al., 2005; Lamy et al., 2011), and importantly, the effects can differ from those that target changes have at the level of early visual selection (e.g., Becker, 2010b, 2013). As Becker (2013) recently showed in a shape search task, manual RT can show strong priming effects when the target is presented in a former non-target shape (or vice versa), whereas the first eye movements to the target is not at all affected by these half-switches. These results highlight that RT switch costs do not always indicate switch costs in visual selection. Therefore, studies on priming should not rely exclusively on manual RT, but include other, location-based measures that allow more direct inferences about visual selection (e.g., McPeek et al., 1999; Becker, 2008a,b,c,2010a, 2013).

## EXPERIMENT

In the present experiment, the observer’s task was to search for a pop-out target that had a different color than the non-targets, and to respond to an additional item inside the target. To test the relational account against feature-based accounts, we varied the color of the target and/or the non-targets so that either the relative color, the absolute color, or both the relative and absolute color of the target always remained constant (in blocked conditions). Moreover, to ensure generalizability of the results, two different sets of colors were used: the first color set comprised the similar colors red-orange, orange, and yellow–orange. The second set of colors was dissimilar and comprised full red, orange, and full yellow.

**FIGURE 1:**
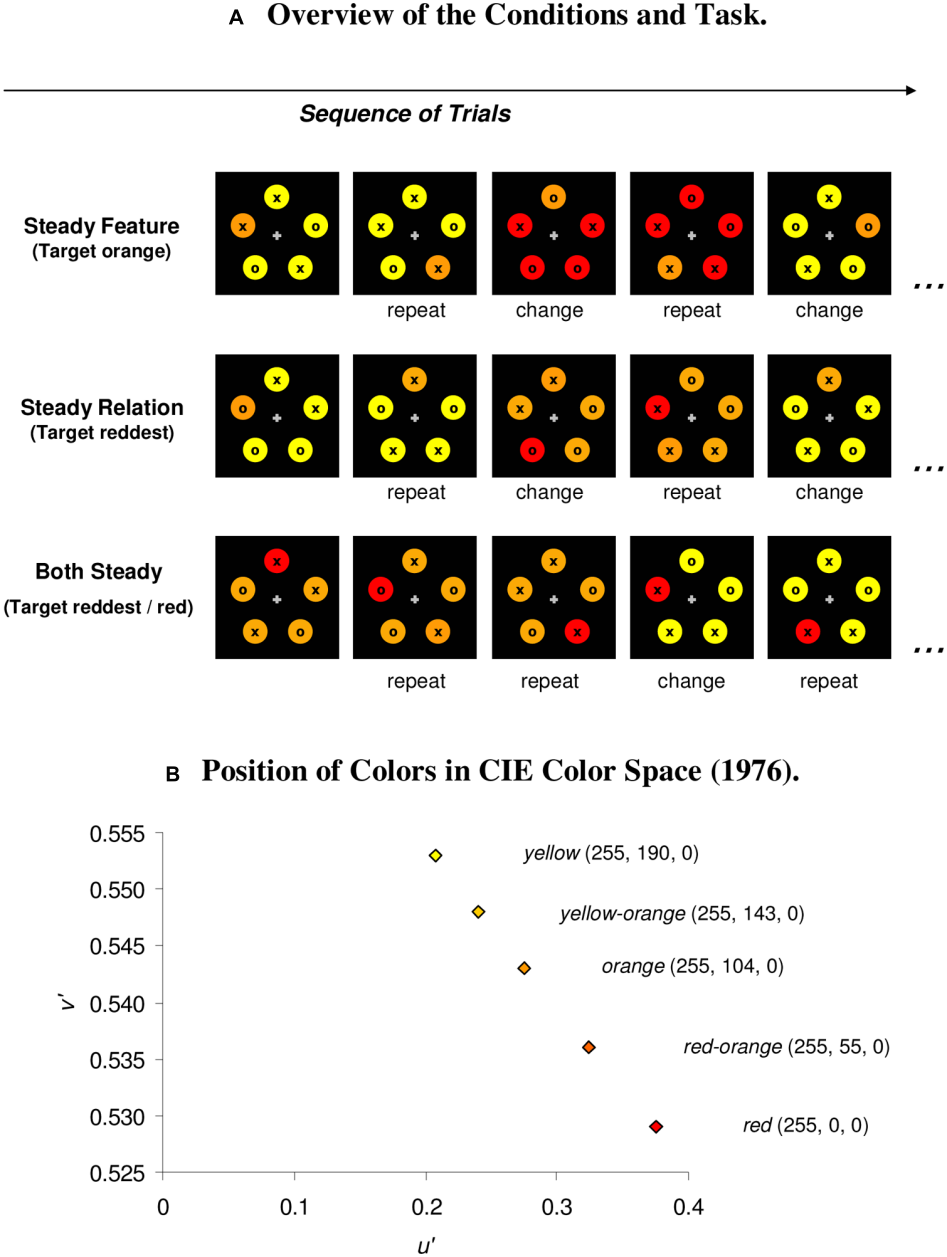
**(A) The participant’s task in the experiment was to search for the odd colored target and to respond to the item inside the target with a button press (o/x: right/left mouse button).** Priming effects were assessed across three blocked conditions, in which the target’s color was kept constant (orange; steady feature condition), its relative color was constant (redder; steady relation condition), or both attributes were constant (both steady condition). **(B)** RGB values for the colors and position of colors in CIE color space (1976).

Search performance was assessed in three blocked conditions: first, in a *steady feature condition*, the target’s color value always remained the same (encouraging single channel processing), but on switch trials, the non-targets changed such that the relative color of the target changed (from redder to yellower, or vice versa). For instance, in the similar color condition, the target was orange, and the non-targets could be either all yellow–orange or all red-orange. In the *steady relation condition*, both the target and non-target colors changed on switch trials such that the non-targets inherited the previous target color on switch trials or *vice versa*, but the relative color of the target always remained the same (e.g., target redder). For instance, with the dissimilar colors, search displays consisted of a red target among orange non-targets, or of an orange target among yellow non-targets. Finally, there was a baseline condition (*both steady condition*), in which both the target’s color value and its relative color were constant and only the non-target color changed, in a way that the relative color of the target was unaffected. For instance, with the similar colors, the target was always red-orange, and the non-targets could be either all orange or all yellow–orange (target always redder; see **Figure 1**).

According to feature-based accounts of priming, the target and non-target colors are processed in separate channels, and changing the target color or the non-target color can each produce switch costs, which are however, independent of each other (i.e., additive costs; e.g., Maljkovic and Nakayama, 1994; Martinez-Trujillo and Treue, 2004; Navalpakkam and Itti, 2006; Kristjansson and Driver, 2008; Lamy et al., 2008). Hence, the feature priming account predicts that changing only the non-target color value (as in the steady feature condition and the both steady condition) should produce lower switch costs than changing both the target and the non-target color value (as in the steady relation condition). This should at least hold when the amount of color change is the same across conditions, which is the case when we compare the similar color sets of the steady feature condition (yellow–orange, red-orange) with the dissimilar color sets of the steady relation condition and the both steady condition (yellow, orange).

A relational account of priming would predict that switch costs mainly occur when the *relative* color of the target changes. Hence, switch costs should occur only in the steady feature condition, in which the target’s relative color changes from redder to yellower (or vice versa), not in the steady relation condition or the both steady condition, in which the relative color of the target is constant (target always redder). Table 1 provides an overview of the inter-trial changes in each condition and the predicted results.

**Table 1:** Overview of conditions and predictions for experiment 1.

Condition	Manipulation	Constant	Predicted switch cost		Results
			Relational account	Feature-based account
Steady feature/Relation varies	Only non-targets change, relation varies	Target color (*orange*)	Switch cost	No switch cost (or small switch cost)	Switch cost
Steady relation/Feature varies	Target and non-targets change, relation remains the same	Relative target color (*redder*)	No switch cost	Switch cost (large)	No switch cost
Both steady *baseline*	Only non-targets change, relation remains the same	Target color and relative target color	No switch cost	No switch cost (or small switch cost)	No switch cost

*Overview of the conditions and predictions of the present study. The conditions are described in the three left-most columns, with the middle column specifying which item remained constant in each condition. The columns to the right list the predictions for the relational account and a feature-based account, with the right-most column providing a description of the results.*

As described above, we assessed priming effects on search performance by analyzing the effects of color priming on the mean search times – that is, the time from the onset of the search display to the point in time in which the eyes are fixating on the target (for the first time in a trial). Moreover, to examine effects that are present at the very beginning of a trial, we also report the proportion of trials in which the first eye movement went directly to the target and the mean latencies of these fixations (see also Becker, 2010a, 2013).

### METHODS

#### Participants

Twelve volunteers from the University of Queensland (8 female, 4 male) participated in the experiment (mean age, 23.1; range, 18–33). All participants were paid $10 for their participation, were naïve as to the purpose of the experiment, and had normal or corrected-to-normal vision. Informed consent was obtained from all participants.

#### Materials

A personal computer with a 2.4 GHz Intel Core 2 Duo CPU and a 21-in. color LCD monitor (BenQ FP92V) was used to produce and display the stimuli. Stimuli were presented with a resolution of 1,280 × 1,024 pixels and a refresh rate of 75 Hz. For eye tracking, a video-based infra-red eye tracker with a spatial resolution of 0.1° and a temporal resolution of 500 Hz was used (EyeLink 1000, SR Research Ltd., ON, Canada). Participants were seated in a well-lit room, with their head fixated by the eyetracker’s chin rest and forehead support, and viewed the screen from a distance of 63 cm. For registration of manual responses, a standard USB optical mouse was used. Event scheduling and RT measurement were controlled by the Presentation software (Neurobehavioral Systems).

#### Stimuli

All stimuli were presented against a white background (50.4 cd/m^2^). The fixation display consisted of a small black fixation cross (0.2° × 0.2°) presented at the center of the screen. Search displays consisted of five colored disks (diameter: 1.25°) that were placed equidistantly on the outlines of an imaginary circle with a diameter of 13.4°, beginning at the 12o’clock position. Two sets of colors were used: the similar colors contained the colors red-orange, orange, and yellow–orange. The dissimilar colors comprised full red, orange, and yellow (see **Figure 1B** for the RGB values and positions in CIE color space). The response-defining stimuli consisted of small black “o” or “x” characters (0.2° × 0.2°; Arial Black), which were located at the center of the colored disks. Feedback displays consisted of the black printed words “right” or “wrong” (Arial) and were presented at the center of the screen.

#### Design

The experiment consisted of six blocked conditions; three conditions each for the similar color set and the dissimilar color set. In the steady feature condition, the target was always orange, and the non-targets varied between yellow–orange and red-orange (similar colors) or yellow and red (dissimilar colors). In the steady relation condition (i.e., target always redder), the similar target could be either red-orange among orange non-targets, or orange among yellow–orange non-targets. The dissimilar target was either red among orange non-targets, or orange among yellow non-targets. In the both steady condition, the similar target was always red-orange, and presented among either orange or yellow–orange non-targets. The dissimilar target was always red, and was presented among either orange or yellow non-targets (see **Figure 1**, **Table 1** for an overview of the conditions).

The target position, target and non-target colors and the response-defining item were chosen randomly on each trial, with the limitation that each display contained an equal number of left and right response-indicative items (exempting the target). The probability for repeat trials was 50%.

#### Procedure

Each trial started with the presentation of a small black fixation cross and a fixation control: the search display was only presented if the tracking was stable (no blinks) and the gaze was within 0.8° of the center of the fixation cross, for at least 500 ms (within a time-window of 2 s). Otherwise, participants were calibrated anew (9-point calibration) and the next trial started again with the fixation control.

Upon presentation of the stimulus display, the fixation cross disappeared and participants could make an eye movement to the pre-defined target. Upon finding the target, participants had to press the right mouse button if the response-indicative item inside the target square was an “o,” and the left mouse button when it was an “x.” The stimulus display remained on screen until response, and was immediately succeeded by the feedback display that informed participants whether the manual response had been correct or wrong. After an inter-trial interval of 250 ms, in which a blank white screen was presented, the next trial started with the presentation of the fixation cross.

Prior to the experiment, participants were given written instructions about the task. The instructions emphasized that the eye movement to the target should be made as fast and as accurately as possible, whereas the manual response should be made as accurately as possible. Moreover, prior to each block, participants were given detailed instructions about the possible target and non-target colors, and were provided with examples of both displays. The instructions emphasized that the target color would always be the same in the steady feature condition and the both steady condition, and informed participants that the target and non-target colors would change in the steady relation condition, though the target would always be redder than the non-targets. Each blocked condition consisted of 150 trials (900 trials in total). On average, it took 50 min to complete the experiment.

## RESULTS

### DATA

Eye movements were parsed into saccades, fixations, and blinks using the standard parser configuration of the EyeLink 1000 software, which classifies an eye movement as a saccade when it exceeds a velocity of 30°/s or an acceleration of 8,000°/s^2^. The first eye movement on a trial was attributed to the target when the saccade ended within 1.6° of the center of the target. Fixation latencies were computed from the onset of the trial to the point in time where the first saccade landed on the target (as per velocity/acceleration criterion).

Trials were excluded from all analyses when the target had not been selected within 1 s from the onset of the search display. This led to a loss of 0.4% of all data. In addition, trials with a manual error were excluded from the analysis, which led to a further loss of 2.9% of the data.

### SEARCH TIME

**FIGURE 2:**
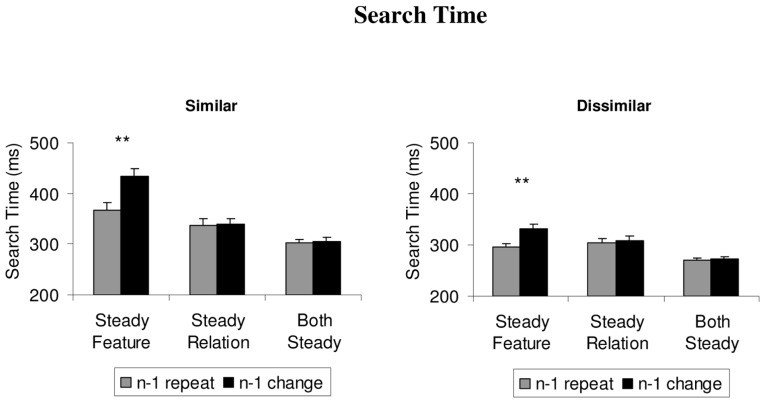
**The mean search times (from the onset of the search display to the first fixation on the target), depicted separately for similar and dissimilar colors in the steady features, steady relations, and both steady conditions.** Feature priming effects are depicted as the difference between repeat trials (gray histograms) and switch trials (black histograms). Error bars depict +1 SEM. **p < 0.01.

The mean search times for each of the conditions are depicted in **Figure 2**. For statistical analysis of the data, we first computed two separate 3 × 2 ANOVAs over the mean search times of the similar and the dissimilar color sets, respectively. Each analysis comprised the within-subject factors condition (steady feature, steady relation, both steady) and repetition (repeat trial, change trial). For the similar colors, the results showed a significant main effect of the condition, *F*(2,22) = 48.3, *p* < 0.001, $\eta_{p}^{2}$ = 0.81, repetition, *F*(1,11) = 48.3, *p* < 0.001, $\eta_{p}^{2}$ = 0.79, and a significant interaction between the variables, *F*(2,22) = 56.8, *p* < 0.001, $\eta_{p}^{2}$ = 0.84. Two-tailed *t*-tests showed that switch costs, with slower search on (non-target) change trials than (non-target) repeat trials, were confined to the steady feature condition, *t*(11) = 10.4, *p* < 0.001, and did not occur in the both steady condition or on (target) change trials compared to (target) repeat trials in the steady relation condition, *t’*s < 1.

For the dissimilar colors, the results were very similar: the 3 × 2 ANOVA showed significant main effects of condition, *F*(2,22) = 22.0, *p* < 0.001, $\eta_{p}^{2}$ = 0.66, and repetition, *F*(1,11) = 48.2, *p* < 0.001, $\eta_{p}^{2}$ = 0.81, as well as a significant interaction between the variables, *F*(2,22) = 15.9, *p* = 0.001, $\eta_{p}^{2}$ = 0.59. Follow-up *t*-tests showed that switch costs occurred only in the steady feature condition, *t*(11) = 5.8, *p* < 0.001, not in any of the other conditions, *t’*s < 1.9, *p’*s > 0.07.

### FIRST TARGET FIXATIONS

**FIGURE 3:**
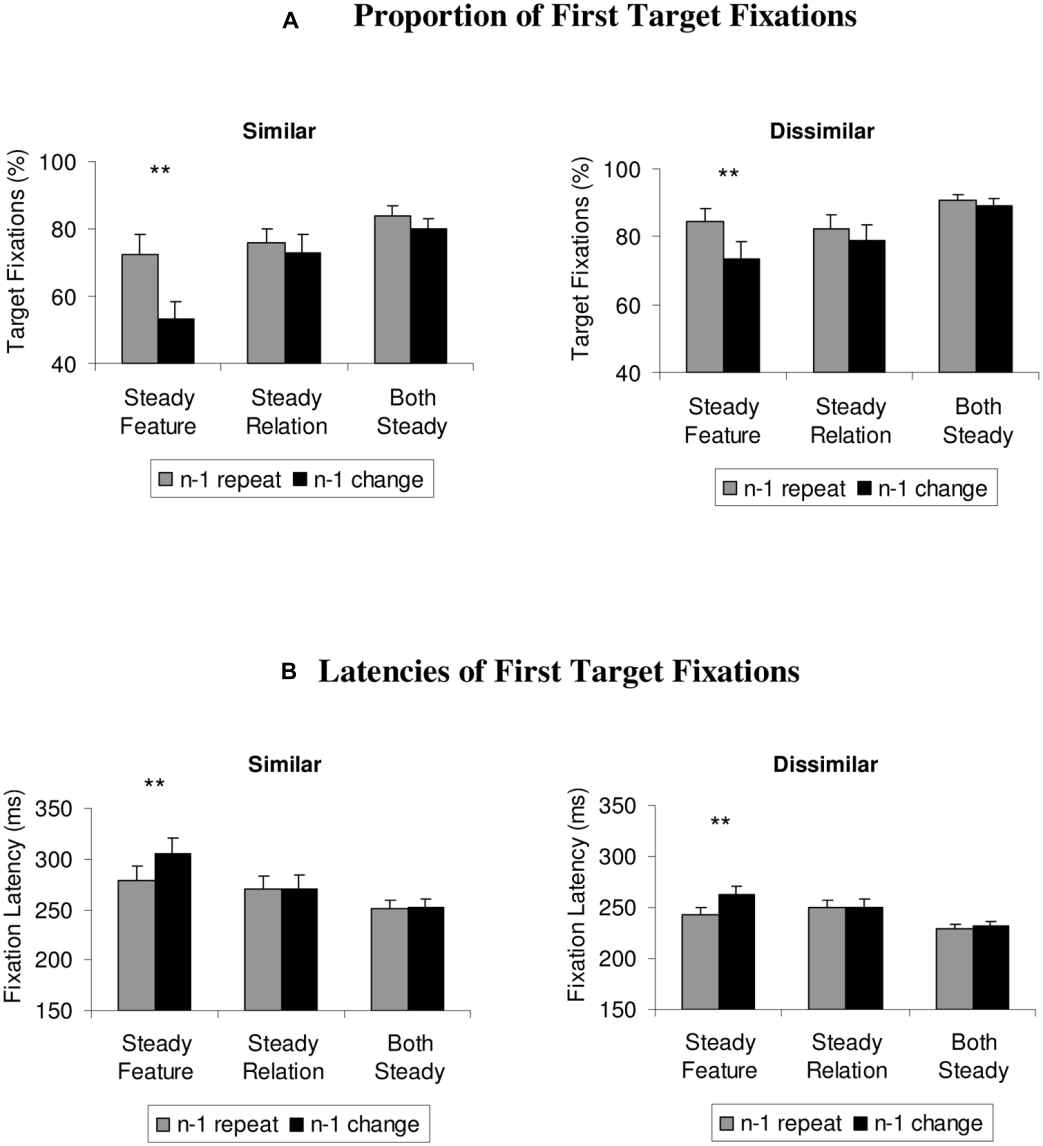
**(A)** Proportion of first target fixations and **(B)** latencies of first target fixations, depicted separately for steady features, steady relations, and steady features and relations (both steady), for similar and dissimilar target and non-target colors. Feature priming effects are depicted as differences between repeat trials (gray histograms) and switch trials (black histograms), and were limited to the steady feature condition. Error bars depict +1 SEM. **p < 0.01.

To examine whether the effects observed in the mean search times were already present at an early stage of visual search, the same analysis was computed over the proportion and latencies of first eye movements that directly went to the target (see **Figure 3**). One participant had to be excluded from these analyses, because in some of the conditions, he selected the target only on 2% of all trials as the first item. For the similar color set, the 3 × 2 ANOVA showed a significant main effect of the condition, *F*(2,20) = 17.9, *p* < 0.001, $\eta_{p}^{2}$ = 0.64, repetition, *F*(1,10) = 64.9, *p* < 0.001, $\eta_{p}^{2}$ = 0.87, and a significant interaction between the variables, *F*(2,20) = 10.3, *p* = 0.001, $\eta_{p}^{2}$ = 0.51. Two-tailed *t*-tests showed that switch costs occurred only in the steady feature condition, *t*(10) = 6.5, *p* < 0.001 (other *t’*s < 2.1; *p’*s > 0.06).

For the dissimilar colors, the analysis showed similar results, with a significant main effect for the condition, *F*(2,20) = 9.2, *p* = 0.002, $\eta_{p}^{2}$ = 0.48, repetition, *F*(1,10) = 14.6, *p* = 0.003, $\eta_{p}^{2}$ = 0.59, and a significant interaction, *F*(2,20) = 4.4, *p* = 0.041, $\eta_{p}^{2}$ = 0.31. Follow-up *t*-tests revealed that significant switch costs occurred only in the steady feature condition, *t*(10) = 3.4, *p* = 0.006, not in the other conditions, *t’*s < 2.0, *p’*s > 0.08.

### LATENCY OF FIRST TARGET FIXATION

To ensure that the results from the first target fixations were not contaminated by a speed-accuracy trade-off, the latencies of the first eye movements to the target were analyzed in the same way (see **Figure 3B**). The 3 × 2 ANOVA computed over the data from the similar color set showed a similar results pattern as observed in the first fixations, with significant main effects of the condition, *F*(2,20) = 16.3, *p* = 0.001, $\eta_{p}^{2}$ = 0.62, repetition, *F*(1,10) = 7.2, *p* = 0.023, $\eta_{p}^{2}$ = 0.42, and a significant interaction, *F*(2,20) = 6.9, *p* = 0.008, $\eta_{p}^{2}$ = 0.41. Two-tailed *t*-tests confirmed that switch costs were found in the steady feature condition only, *t*(10) = 3.0, *p* = 0.013 (other *t’*s < 1).

The analysis of the dissimilar color set was also in line with the previous findings, showing significant main effects of condition, *F*(2,20) = 14.4, *p* < 0.001, $\eta_{p}^{2}$ = 0.59, repetition, *F*(1,10) = 26.1, *p* < 0.001, $\eta_{p}^{2}$ = 0.72, and a significant interaction, *F*(2,20) = 5.5, *p* = 0.027, $\eta_{p}^{2}$ = 0.36. Two-tailed *t*-tests confirmed that switch costs were again restricted to the steady feature condition, *t*(10) = 4.7, *p* = 0.001 (other *t’*s < 1.7, *p’*s > 0.12).

### COMPARING SWITCH COSTS ACROSS CONDITIONS

Across all dependent variables (search time, first fixations to target, fixation latencies) and the two color sets (similar, dissimilar), the results showed that large and significant switch costs were limited to the steady feature condition, in which only the non-target colors changed. However, one of the other conditions (steady relation or both steady condition) often showed small switch costs that bordered on statistical significance. Thus, we additionally computed the difference scores between switch trials and repeat trials, for all dependent measures and conditions, and tested whether switch costs observed in the steady feature condition would also be significantly larger than in either of the two other conditions. The results showed that, in both conditions (similar, dissimilar) and for all dependent measures (search time, first fixations to target, fixation latencies), switch costs observed in the steady feature condition were significantly larger than the non-significant switch costs in the steady relation condition (all *t’*s > 2.4, *p’*s < 0.037), and the both steady condition (all *t’*s > 2.3, *p’*s < 0.043), with the only exception of non-significant differences in the proportion of first target fixations in the dissimilar condition between the steady relation and steady feature condition, *t*(10) = 2.1, *p* = 0.060). More strikingly perhaps, the steady relation condition and the both steady condition never showed any differences in the non-significant priming effect (all *t’*s < 1). These results clearly show that reliable switch costs were found only upon reversals of the target’s relative color, not with any other changes of the target color or the non-target color.

## DISCUSSION

The results clearly showed that switch costs occurred only in the steady feature condition, in which only the non-target colors changed across trials (e.g., between yellow and red), but not in the steady relation condition, in which both the target and non-target colors changed across trials. These results support the relational account and are inconsistent with a feature-based account. In advance to previous studies, the present results confirmed that processing the target via a single channel does not eliminate feature priming effects: in the steady feature condition, the target was always orange. According to feature-based accounts, the target was thus always processed via the same channel. Yet, changing only the non-target color produced significant switch costs. These switch costs cannot be attributed to the change in the non-target color (e.g., non-target inhibition) because changing the non-target colors failed to produce significant switch costs in the steady relation condition and the both steady condition.

In advance to previous studies (Becker, 2010a), the lack of significant switch costs in the steady relation condition cannot be attributed to the colors being more similar or the color changes being smaller in one condition: in the steady feature condition that yielded significant switch costs, the non-targets changed between yellow–orange and red-orange in the similar color set. As shown in **Figure 1B**, the amount of color change was the same in the steady feature condition with the dissimilar color set, in which the colors changed from orange to yellow. Moreover, as shown in **Figures 2** and **3**, the results were qualitatively the same for large and small color changes (i.e., similar, dissimilar colors) within each condition, effectively ruling out an explanation in terms of color similarity or the amount of color change involved in any of the conditions.

Note also that the present set of conditions (steady feature, steady relation, both steady) allowed a decisive test of feature-based accounts. Feature-based accounts centrally claim that the target color and the non-target color are processed independently and separately from each other. With this, priming effects can only result from carry-over effects of target activation (i.e., enhancing the gain of the target feature) or non-target inhibition (i.e., a reduced gain of the non-target feature). However, both target activation and non-target inhibition should have produced switch costs in the steady relation condition, because in this condition, the target and the non-target colors both changed on switch trials, such that the target either inherited the previous (inhibited) non-target color, or the non-targets inherited the previous (activated) target color. According to feature-based accounts, this condition should definitely have shown significant switch costs, contrary to the results. The fact that the steady relation condition failed to show any switch costs clearly demonstrates that the target and non-target colors are not processed (or weighted) independently of each other, in line with the relational account.

In addition, no current feature-based theory can explain the finding of larger switch costs in a condition in which only the non-targets change (steady feature) than in a condition in which both target and non-targets change, in the form of a half-switch (steady relation condition). With this, the present set of results provides strong evidence against feature-based accounts of priming.

## GENERAL DISCUSSION 

The present study provided a more decisive test of the relational account versus a feature-based account of color priming. The results clearly showed that the target and non-target colors are not processed independently of each other – contrary to a central tenet of current feature-based theories of attention (e.g., Treisman and Sato, 1990; Wolfe, 1994; Lee et al., 1999), and priming effects (e.g., Maljkovic and Nakayama, 1994). In particular, the results of the present study support the view that priming effects are based on relative features, by showing that (1) switch costs occur only when the relative color of the target changes, not when the physical color of the target or the non-targets change; (2) that priming affects visual search at an early stage of visual selection (see results from the first eye movements on a trial; e.g., McPeek et al., 1999; Goolsby and Suzuki, 2001; Bichot and Schall, 2002; Becker, 2008a,b,c, 2010a, 2013; Eimer et al., 2010). The results moreover extend on previous results by showing that (1) color priming effects were largely independent on whether or not colors were similar or dissimilar, and that (2) switch costs occurred despite the fact that the target color never changed, i.e., that the target could in principle be found by biasing selection to a single channel (according to feature-based accounts). With this, the study adds to the growing body of evidence that visual selection operates on relative features, not physical features (e.g., Becker, 2010a, 2013; Becker et al., 2010, 2013)

### ROLE OF STRATEGIC FACTORS

One aspect of the present design worth considering is that the target and/or non-targets changed differently in the three blocked conditions, thus allowing observers to adopt different top-down search strategies. Keeping the target color value constant in the steady feature condition was deemed necessary for a stringent test of feature-based accounts, because observers can only be expected to (try to) bias visual selection to a particular color value if the target color value does not change. However, it could still be asked whether the differences in the priming effect could be ultimately due to strategic factors.

For instance, if it were easier to search for a relative color than a specific physical color, then could not switch costs arise because it takes longer to adopt a search template for a specific color value (i.e., orange) rather than for a relative color (e.g., redder)?

**FIGURE 4:**
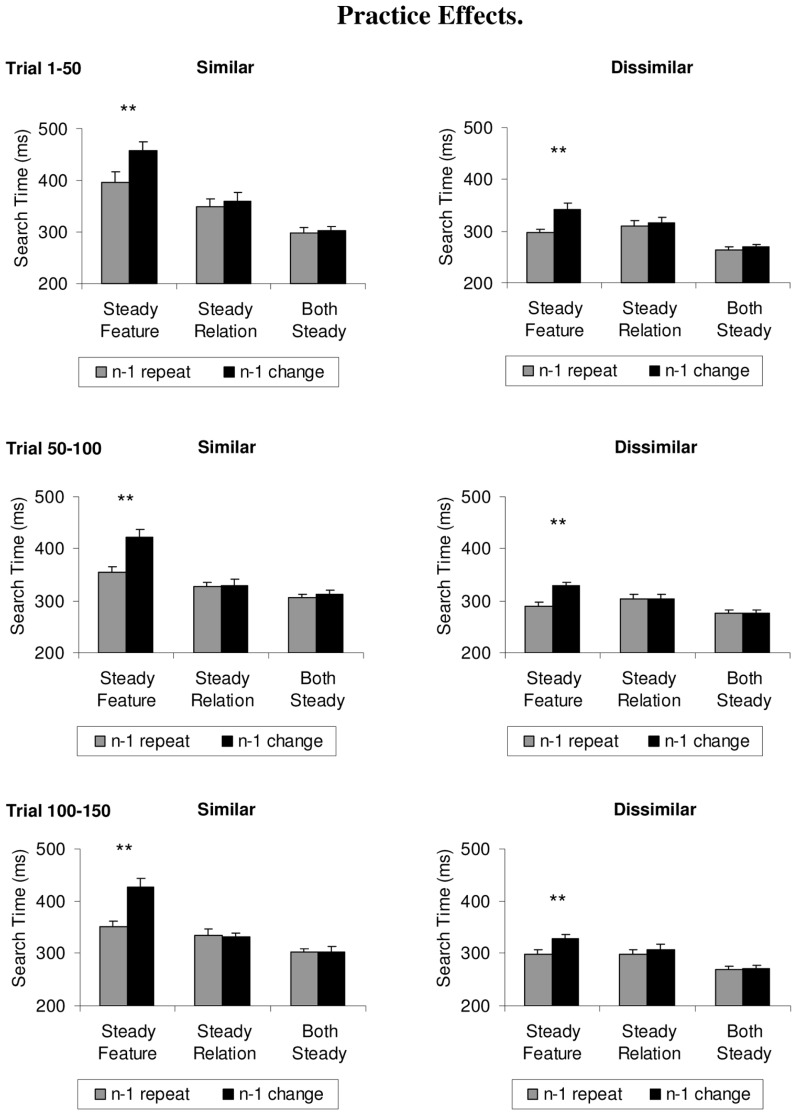
**Effect of practice on search times, depicted separately for the similar and dissimilar color sets in the steady features, steady relations, or both steady conditions, for the first, second, and third 50 trials (top to bottom).** Feature priming effects are shown as difference between repeat trials (gray histograms) and switch trials (black histograms), and did not vary as a function of practice. Error bars depict +1 SEM. **p < 0.001.

To address this question, we analyzed search times separately for the first, second, and third 50 trials in each block, separately for each condition (see **Figure 4**). 3 × 2 ANOVAs comprising the within-subject factors practice (first 50 trials, second 50 trials, third 50 trials) and repetition (repeat trials, change trials) were first computed over the search times of the similar colors. The steady feature condition showed a significant main effect of practice, *F*(2,22) = 10.9, *p* = 0.004, $\eta_{p}^{2}$ = 0.50, and of repetition, *F*(1,11) = 129.0, *p* < 0.001, $\eta_{p}^{2}$ = 0.92, but no significant interaction between the variables, *F* < 1. The steady relation condition showed only a significant main effect of practice, *F*(2,22) = 4.7, *p* = 0.029, $\eta_{p}^{2}$ = 0.30 (all other *F’*s < 1), whereas the both steady condition showed no significant main effects or interactions, *F’*s < 1.3, *p’*s > 0.27.

The search times for the dissimilar colors failed to show a significant practice effect in the steady feature condition, *F*(2,22) = 3.1, *p* = 0.07, the steady relation condition, *F*(2,22) = 1.5, *p* = 0.25, and the both steady condition, *F*(2,22) = 1.6, *p* = 0.22. Similarly, the interactions were non-significant (the only significant effect being a significant main effect of repetition in the steady feature condition, *F*(1,11) = 34.8, *p* < 0.001, $\eta_{p}^{2}$ = 0.76).

The analysis of color priming over time fails to support the notion that priming effects in the steady feature condition could be attributed to differences in the time-course of adopting an appropriate search strategy. Practice effects never modulated the priming effect. Indeed, priming effects were absent from the start of the experiment and throughout for the steady relation condition and the both steady condition, and present from the start to the end for the steady feature condition. Hence, we can conclude that the differences in the priming effect were not due to differences in the time-course of adopting the appropriate target template^1^.

### CHANNELS AND THE RELATIONAL ACCOUNT

It could be argued that the results of the present study are still consistent with a feature-based account, if we assume that both the similar and dissimilar colors were processed via two broad and largely overlapping channels (e.g., Wolfe, 1994). In this case, red, red-orange and orange would all have been covered by a “red” channel, with the activation produced by each color systematically decreasing from red over red-orange to orange. Visual selection of the redder target would have relied on the target producing a higher activation in this channel than the yellow–orange or yellow non-target colors, and vice versa for selection of the yellower target and the “yellow” channel (which would respond more strongly to yellow than yellow–orange and orange). A two-channel account with such a “relational” selection rule would indeed seem consistent with the data. However, such a two-channel account would not be noticeably different from the relational account and thus, not at odds with a relational view.

Of note, the relational account does not deny that there are channels for color processing – in fact, color-specific processing channels have been discussed at length as a possible substrate for biasing attention to relative colors (e.g., Becker, 2010a, 2013). What the relational account centrally claims is that visual selection of a “basic” feature such as a clearly discriminable color depends on other (nominally irrelevant) colors in the context. Or, in other words, what is denied is that target and non-target features are processed independently of each other in separate channels, which is a central assumption in attention research in general, and feature priming effects specifically (e.g., Maljkovic and Nakayama, 1994; Wolfe, 1998; Lee et al., 1999; Lamy et al., 2008). It is easy to see that a two-channel account would violate this separate processing assumption, because the orange target would be selected via a red channel or a yellow channel *depending on the context colors*. A context-dependent selection bias is exactly what the relational account claims. Hence, in the context of the present experiment, a two-channel account can be regarded as a possible implementation of the relational account and is not at conflict with it. Similarly, the relational account would be consistent with largely independent encoding of the categorical colors (blue, red, yellow, green), because these do not have a clearly defined relationship over and above their properties of being bluer, redder, yellower, and greener (e.g., Ansorge and Becker, 2014).

One central disadvantage of the categorical channel accounts (e.g., Wolfe, 1994) is that they do not allow efficient selection of mixture colors when there are multiple colored items. For instance, a two-channel account could not explain efficient selection of an orange target when it is embedded among multiple red and yellow non-target items. This holds because, in the absence of a dedicated orange channel, the red and yellow non-targets would produce higher activations in the red and yellow channels than the orange target, so that the orange target could not be located pre-attentively (e.g., Wolfe, 1994). In contrast to this prediction, it has been shown that an orange item can be successfully located among red and yellow non-targets (e.g., Bauer et al., 1995; Harris et al., 2013; Becker et al., 2014). In fact, Becker et al. (2013) showed that a briefly presented orange cue among two red and two yellow other cues can involuntarily capture attention, showing that orange *is* pre-attentively localizable among red and yellow.

These results would appear to be inconsistent with a two-channel account and point to the existence of a dedicated orange channel (e.g., D’Zmura, 1991; Lee et al., 1999; Navalpakkam and Itti, 2006). If we however have a dedicated orange channel, then why was it not used in the steady feature condition to bias selection to orange while excluding red and yellow? – it is questions like these that current feature-based accounts have not addressed (e.g., Wolfe, 1994; Navalpakkam and Itti, 2006; Meeter and Olivers, 2014).

According to the relational account, the failure to obtain more evidence for the orange channel in this and other experiments is due to the fact that there is a strong preference in the visual system to bias visual selection to the relative color of the target instead of its physical color (e.g., Becker, 2010a; Becker et al., 2013, 2014; Harris et al., 2013). With this, the relational account seems to provide a better explanation for present and past results, because it avoids the need to constantly re-define the number of channels and their bandwidth so that they match the results.

## CONCLUSION

The present study critically tested whether color priming effects depend on the relative color of the previous and current target, or on the physical colors of the target and non-targets. To that end, we measured the observer’s eye movements in pop-out search among similar and dissimilar colors, when either only the relative target color or only its absolute color remained constant. The results clearly supported the relational account, by showing that visual selection was primed toward the relative color of the target. Additional analyses moreover showed that visual selection was biased toward the relative target color from the beginning of the experiment to the end. These results show, for the first time, that relative colors were primed from the beginning of the experiment and persisted throughout the task. These results refute the prevalent view that elementary features such as colors are encoded separately and independently of each other, and instead support a context-dependent account of color priming.

## Conflict of Interest Statement

The authors declare that the research was conducted in the absence of any commercial or financial relationships that could be construed as a potential conflict of interest.

## Footnotes

1 It is still possible that different top-down strategies were adopted early within each blocked condition and modulated the priming effect. However, no easy mapping exists between top-down conditions and priming effects, because the target always remained constant in two conditions, one of which showed priming effects (steady feature condition), whereas the other one failed to show priming effects (both steady condition).
